# Clinical outcomes of patients treated with intravenous zanamivir for severe influenza A(H1N1)pdm09 infection: a case report series

**DOI:** 10.1186/s12879-019-4530-1

**Published:** 2019-10-16

**Authors:** Carlo Torti, Maria Mazzitelli, Federico Longhini, Eugenio Garofalo, Andrea Bruni, Aida Giancotti, Giorgio Settimo Barreca, Angela Quirino, Maria Carla Liberto, Francesca Serapide, Giovanni Matera, Enrico Maria Trecarichi, Paolo Navalesi, the IMAGES (Integrated MAnaGEment of Sepsis) Group, Vincenzo Pisani, Chiara Costa, Giuseppe Greco, Vincenzo Scaglione, Rosaria Lionello, Valentina La Gamba, Eugenio Biamonte, Ovidia La Valle, Giuseppina Cimino, Paola La Torre, Abdalla Karim, Antonio Gemelli

**Affiliations:** 1 Department of Medical and Surgical Sciences, Infectious and Tropical Diseases Unit, “Magna Graecia” University of Catanzaro, Catanzaro, Italy; 2 Department of Medical and Surgical Sciences, Unit of Intensive Care, “Magna Graecia” University, Catanzaro, Italy; 3 Department of Health Sciences, Unit of Clinical Microbiology, “Magna Graecia” University, Catanzaro, Italy

**Keywords:** Zanamivir, Influenza A(H1N1)pdm09, ECMO, ICU

## Abstract

**Background:**

Intravenous (IV) zanamivir could be a suitable alternative for the treatment of severe influenza A(H1N1)pdm09 infection in patients who are unable to take oral or inhaled medication, for example, those on mechanical ventilation and extracorporeal membrane oxygenation (ECMO). However, data on the clinical outcomes of such patients is limited.

**Case presentation:**

We report the clinical outcomes of four patients who were admitted at the intensive care unit during the 2017–2018 influenza season with severe sepsis (SOFA score > 11) and acute respiratory distress syndrome requiring ECMO and mechanical ventilation. Two patients were immune-compromised. The A(H1N1)pdm09 genome was confirmed by polymerase chain reaction (PCR) on nasopharyngeal specimen swabs prior to administration of IV zanamivir at a dose of 600 mg twice daily. Weekly qualitative PCR analysis was done to monitor viral clearance, with zanamivir treatment being discontinued upon receipt of negative results. In addition, the patients were managed for concomitant multidrug-resistant bacterial infections, with infection resolution confirmed with blood cultures.

The median time for zanamivir treatment was 10 days (IQR 10–17). The clinical outcome was favourable with all four patients surviving and improving clinically. All four patients achieved viral clearance of A(H1N1)pdm09 genome, and resolution of multidrug-resistant bacterial infections.

**Conclusions:**

IV zanamivir could be a good therapeutic option in patients with severe influenza A(H1N1)pdm09 infection who are unable to take oral or aerosolised antiviral medication. We recommend prospective randomized control trials to support this hypothesis.

## Background

Influenza due to the 2009 pandemic A/H1N1 virus, abbreviated as A(H1N1)pdm09, a viral disease of public health concern, causes between three to 5 million cases globally, frequent hospitalizations and over 650,000 deaths annually [1, 2]. Seasonal influenza is one of the leading causes of admissions to intensive care units (ICU) due to acute respiratory distress syndrome (ARDS), sometimes requiring extracorporeal membrane oxygenation (ECMO) support [3].

Oseltamivir is the first-line treatment for A(H1N1)pdm09 infection and is administered orally [4]. However, the pharmacokinetic properties and concerns for resistance [5–7], make it unsuitable in patients with severe life-threatening infection and are unable to take oral medication. The bioavailability of oseltamivir has not been extensively studied in patients who are on mechanical ventilation and feed via a nasogastric tube. A small study investigated its pharmacokinetic effects only for three patients with severe H5N1 influenza [8]. Therefore, other therapeutic strategies, such as intravenous (IV) use of zanamivir, may be of benefit. An international, phase 3 randomized control trial, demonstrated that IV zanamivir had similar antiviral efficacy and safety profile to oral oseltamivir in patients with severe influenza infection [9]. Moreover, time to clinical response did not differ between 600 mg and 300 mg twice daily treatments.

On 28 February 2019, the Committee for Medicinal Products for Human Use (CHMP) adopted a positive opinion, authorizing the marketing of IV zanamivir under exceptional circumstances [10, 11]. It is indicated for the treatment of complicated and potentially life-threatening influenza A or B virus infection in adult and paediatric patients (aged ≥6 months) when: (i) the patient’s influenza virus is known or suspected to be resistant to anti-influenza medicinal products other than zanamivir, and/or (ii) other anti-viral medicinal products for treatment of influenza, including inhaled zanamivir, are not suitable for the individual patient.

In this case series, we report the clinical evolution and survival outcome of the largest number of patients who, to the best of our knowledge, underwent ECMO support and were treated with IV zanamivir. Another case report described a single patient who suffered severe respiratory insufficiency was treated with IV zanamivir but was not prescribed ECMO [7].

## Case presentation

During the influenza season in the years 2017–2018, four patients (3 male and 1 female) (aged from 37 to 59 years) were admitted to our ICU for ECMO respiratory support due to severe ARDS related to A(H1N1)pdm09 infection. All patients were transferred from peripheral ICUs for ECMO treatment, after a median hospital length of stay of 9 days (range: 1–10 days). Two patients were immune-compromised; patient #1 due to relapsing Hodgkin lymphoma and patient #2 due to multiple myeloma. The other patients did not suffer from any significant underlying medical conditions.

At ICU admission, all four patients had a sequential organ failure assessment (SOFA) score greater than 11, predicting a mortality rate higher than 50%, while the median acute physiology and chronic health evaluation (APACHE) score was 20 (range: 18–22).

The A(H1N1)pdm09 genome was detected through multiplex polymerase chain reaction (PCR) performed on nasopharyngeal swabs (Biofire**®** FilmArray**®** Respiratory Panel, BioMèrieux diagnostics, Italy), using the procedure previously reported [12]. Besides A(H1N1)pdm09, this test targets a respiratory panel consisting of several viruses (adenovirus, coronavirus 229E, coronavirus HKU1, coronavirus OC43, coronavirus NL63, human metapneumovirus, human rhinovirus/enterovirus, influenza A, influenza A/H1, influenza A/H3, influenza B, parainfluenza 1–4, respiratory syncytial virus) and three bacteria (*B. pertussis*, *C. pneumoniae*, and *M. pneumoniae*). In summary, the test pouch contains all dry reagents required for specimen extraction, first-stage multiplex PCR, and individual second-stage real-time PCRs. Operatively, the pouch was placed in the FilmArray**®** instrument, and a pre-programmed run was initiated. Results were generated using amplification and melting curve data. Negative controls were certified by the manufacturer before the Food and Drug Administration (FDA) and Conformité Européenne (CE) approval of the test. All precautions have been used during the procedure to avoid pre-analytical contamination. The test pouch is a sealed, non-reusable item. Negative controls were run periodically to evaluate the performance of the PCR test using sterile collection media. Weekly nasopharyngeal swabs were taken for PCR qualitative analysis to detect viral clearance and were reported as positive or negative indicating presence or absence of the virus in the swab specimen, respectively. No other viruses were detected in this study and virus isolation was not attempted.

Ethical approval of the study was granted by the local ethics committee for each patient.

IV zanamivir was administered to all four patients on a compassionate basis, at a dosage of 600 mg twice daily [10, 11]. In instances where IV zanamivir was unavailable, oral oseltamivir was administered at a dosage of 75 mg twice daily, to ensure continuity of antiviral treatment for the patients. Antiviral treatment was discontinued when the follow-up PCR results returned negative for A(H1N1)pdm09 genome in the swab specimens. All patients were weaned from ECMO as soon as the intrapulmonary shunt was ≤30%, and mechanical ventilation discontinued after the patient’s ability to breathe spontaneously was assessed [13].

**Fig. 1:**
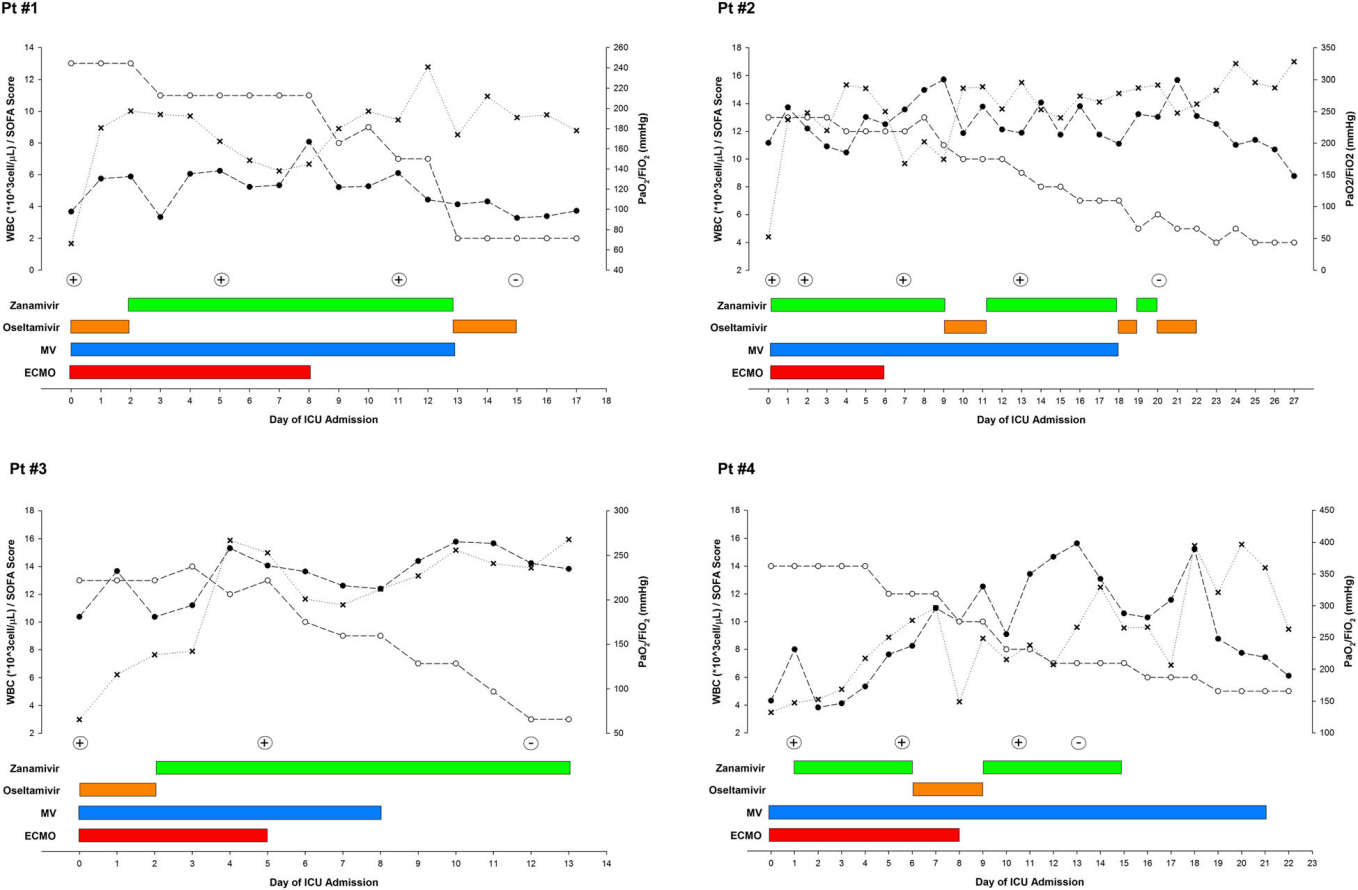
Daily modifications of SOFA, score (empty circles), white blood cell (WBC) count (black circles) and the ratio between arterial partial pressure and inspired oxygen fraction (PaO_2_/FiO_2_) obtained by gas analysis from arterial blood samples (black cross)

Figure 1 depicts, for each patient, daily modifications of the SOFA score, white blood cell count and the ratio between arterial partial pressure and inspired oxygen fraction (PaO_2_/FiO_2_) obtained by gas analysis from arterial blood samples. Table 1 summarises the main results for each patient, including superimposed bacterial infections and antibiotic treatments.

**Table 1:** Main results from individual patients

	Pt #1	Pt #2	Pt #3	Pt #4
Age (years)	47	59	37	58
SOFA score at ICU admission	13	13	13	14
Time spent under ECMO (days)	8	6	5	8
Time spent under MV (days)	13	18	8	21
Day of influenza A (H1N1)pdm09 virus detection	0	0	0	1
Day of first negative influenza A(H1N1)pdm09 virus	15	20	12	13
Length of zanamivir therapy (days)	11	17	11	11
Superinfection by MDR				
Ab	Yes	No	No	Yes
CR-Kp	Yes	Yes	Yes	No
Treatment	colistin+ ceftazidime/avibactam	colistin+ meropenem	colistin+ meropenem+ ertapenem	colistin+ tigecycline+ rifampicin

*SOFA* Sequential Organ Failure Assessment, *ICU* Intensive Care Unit, *ECMO* Extra-Corporeal Membrane Oxygenation, *MV* Mechanical Ventilation, *MDR* Multi-Drug Resistant bacteria, *Ab Acinetobacter baumannii*, *CR-Kp* Carbapenem Resistant *Klebsiella pneumonia*

The median time of ECMO support was 7 days (IQR 6–8 days), while the median time spent under mechanical ventilation was 15 days (IQR 8–21). The median length of stay in the ICU was 20 days (IQR 14–28). The median duration of antiviral therapy was 10 days (IQR 10–17). During treatment, no adverse reactions or side effects were reported. Even though the expected survival of the patients was less than 50% at the time of admission as predicted by SOFA score, clinical conditions progressively improved and all four patients survived.

The clinical conditions of the four patients progressively improved with IV zanamivir and adjuvant therapies despite superimposed multi-drug resistant infections. As summarised in Table 1, patient #1 was diagnosed with concomitant extensively-drug resistant (XDR) *Acinetobacter baumannii* (Ab) pneumonia (susceptible only to colistin) and carbapenem-resistant *Klebsiella pneumoniae* (CR-Kp) and treated with IV colistin and ceftazidime/avibactam for 7 days. Patient #2 had CR-Kp pneumonia and was treated with a two-week course of IV/aerosolized colistin plus meropenem. Patient #3 suffered from CR-Kp pneumonia and secondary bacterial infection and was treated with IV colistin plus double carbapenem (ertapenem and meropenem for 7 days). Patient #4 suffered from XDR-Ab pneumonia with secondary bacteremia and was treated with IV/aerosolized colistin, tigecycline, and rifampicin for 12 days.

All four patients demonstrated both clinical and radiological evidence of resolution of bacterial lung complications. Bloodstream bacterial infection resolution, as defined by no growth of bacteria in the follow-up blood cultures, performed after 5 and 4 days from the previous diagnosis, was confirmed in patients #3 and #4, respectively. Bloodstream infections were determined by detections of bacteria on 3–4 sets of blood cultures, collected from peripheral puncture of one forearm vein of patients under sterile conditions, at the time of fever spikes, chills, leucocytosis and/or septic shock [14]. To exclude the presence of a catheter-related bloodstream infection, a blood sample was also collected directly from the indwelling catheter [14].

## Discussion and conclusions

Despite having severe life-threatening illness because the expected survival of the patients was less than 50% at the time of admission as predicted by SOFA score requiring life support treatment with ECMO, and despite subsequent MDR bacterial superinfections, all four patients at the end of this study survived. This survival could be attributed to the effects of IV zanamivir, although we cannot underestimate the role of the multidisciplinary proactive treatment approach (combined efforts of infectious disease specialists, intensivists and microbiologists) employed in the management of the patients. Moreover, it should be noticed that none of the four cases was treated with zanamivir alone because all received oseltamivir in addition to zanamivir. Therefore, the latter drug contributed to the treatment effect. Although the two drugs were administered separately, they may also have had an additive or synergistic effect in preventing the emergence of A(H1N1)pdm09 isolates resistant to one or the other drug, or treating pre-existing resistant isolates more effectively. Indeed, virological potency of antiviral combination therapy for the treatment of influenza was higher than that of oseltamivir monotherapy, although this did not translate into greater clinical efficacy [15]. Moreover, a study in a mice model showed increased survival in animals infected with A(H1N1)pdm09 virus when treated with oseltamivir-peramivir combination rather than oseltamivir alone [16]. Survival benefit was confirmed in immune-deficient mice infected with A(H1N1)pdm09 influenza virus treated with oseltamivir and favipiravir compared to those treated with either drugs [17]. However, in the same study, oseltamivir resistance was not prevented with this combination treatment [17]. Lastly, clinical efficacy was not superior with oseltamivir-peramivir combination compared with oseltamivir alone in patients with influenza A (H7N9) infection [18]. In conclusion, it is possible that the administration of both drugs for continued antiviral coverage suppressed possible resistant viral strains and increased clinical efficacy but this hypothesis requires further investigations.

Zanamivir proved effective particularly in one patient who was diagnosed with severe pneumonia, impaired gastrointestinal peristalsis and drug malabsorption, requiring life support measures such as deep sedation, neuromuscular blockade and prone positioning [6]. The authors realised viral clearance despite short treatment duration of 10 days and early discontinuation of zanamivir [6].. This finding is similar to another report that described excellent clinical outcomes in two patients who suffered from influenza myocarditis, refractory cardiogenic shock and enteral drug malabsorption, and were treated with IV zanamivir [19]. The authors of the study recommended the use of IV zanamivir if enteral drug malabsorption is suspected and demonstrated (for example, using a paracetamol absorption test) [19].

Some studies have demonstrated the advantage of IV zanamivir over oral oseltamivir. In one study involving mutated viral strains of influenza H1N1 (H274Y mutation), patients who received oseltamivir had prolonged viral shedding and delayed resolution of their symptoms, unlike their counterparts who received zanamivir [20]. This shows that IV zanamivir is effective for oseltamivir-resistant influenza strains. In another study, zanamivir was shown to reduce lung injury in animal models, probably due to its anti-inflammatory properties [21].

A large retrospective report by Chan-Tack et al. described the characteristics of 364 patients who received IV zanamivir, retrieved from the Food and Drug Administration (FDA)’s Emergency Investigational New Drug application database [22]. Among these patients, only 163 (45%) had a confirmed diagnosis of A(H1N1)pdm09 infection of which 74 (20%) received ECMO due to severe ARDS. Although this study did not report specific clinical outcomes in patients receiving ECMO, the authors reported survival in only 38% of patients. In comparison, all patients in our study improved clinically and survived upon receiving IV zanamivir. However, we recognize that the limitations of our study such as small sample size and lack of controls may make this observation difficult to conclude. We recommend large sample size studies to support this hypothesis.

Patient #1 and #2 were immune-compromised. Although this partially explains their prolonged treatments because of virus persistence, it does not seem to offer a similar explanation for patient #3 and #4 who did not have any underlying medical conditions, yet received treatments for relatively prolonged periods of time ranging from 13 to 15 days. In the case of virus persistence and treatment prolongation, virus isolation and running genotypic tests for potential antiviral resistance on neuraminidase as well as molecular monitoring and phenotypic assays are recommended. Indeed, drug-resistance to oseltamivir and zanamivir has been reported in some patients with influenza [23] However, at the end of this treatment course with zanamivir or oseltamivir, all patients achieved clearance of A(H1N1)pdm09 genome in respiratory samples, suggesting that drug resistance was not present or clinically significant.

In conclusion, the findings in our study suggest that IV zanamivir is a good therapeutic option in patients with severe influenza, who are unable to take an oral or aerosolised drug such as those on ECMO and mechanical ventilation. We recommend prospective randomized trials to support this hypothesis as well as pharmacokinetic evaluations and molecular characterisation of influenza viruses to help delineate better prevention and treatment strategies for possible resistant strains.

## Data Availability

All data generated or analysed during this study are included in this published article.

## Abbreviations

APACHE: Acute physiology and chronic health evaluation
CR-Kp: Carbapenem-resistant *Klebsiella pneumoniae*
ECMO: Extracorporeal membrane oxygen
ICU: Intensive care unit
IV: Intravenous
MDR: Multidrug resistance
MV: Mechanical ventilation
SOFA: Sequential organ failure assessment
WBC: White blood cells
XDR: Extensively drug resistance
